# Metabolic flux configuration determination using information entropy

**DOI:** 10.1371/journal.pone.0243067

**Published:** 2020-12-04

**Authors:** Marcelo Rivas-Astroza, Raúl Conejeros

**Affiliations:** Escuela de Ingeniería Bioquímica, Pontificia Universidad Católica de Valparaíso, Valparaíso, Chile; University of Padova, ITALY

## Abstract

Constraint-based models use steady-state mass balances to define a solution space of flux configurations, which can be narrowed down by measuring as many fluxes as possible. Due to loops and redundant pathways, this process typically yields multiple alternative solutions. To address this ambiguity, flux sampling can estimate the probability distribution of each flux, or a flux configuration can be singled out by further minimizing the sum of fluxes according to the assumption that cellular metabolism favors states where enzyme-related costs are economized. However, flux sampling is susceptible to artifacts introduced by thermodynamically infeasible cycles and is it not clear if the economy of fluxes assumption (EFA) is universally valid. Here, we formulated a constraint-based approach, MaxEnt, based on the principle of maximum entropy, which in this context states that if more than one flux configuration is consistent with a set of experimentally measured fluxes, then the one with the minimum amount of unwarranted assumptions corresponds to the best estimation of the non-observed fluxes. We compared MaxEnt predictions to *Escherichia coli* and *Saccharomyces cerevisiae* publicly available flux data. We found that the mean square error (MSE) between experimental and predicted fluxes by MaxEnt and EFA-based methods are three orders of magnitude lower than the median of 1,350,000 MSE values obtained using flux sampling. However, only MaxEnt and flux sampling correctly predicted flux through *E*. *coli*’s glyoxylate cycle, whereas EFA-based methods, in general, predict no flux cycles. We also tested MaxEnt predictions at increasing levels of overflow metabolism. We found that MaxEnt accuracy is not affected by overflow metabolism levels, whereas the EFA-based methods show a decreasing performance. These results suggest that MaxEnt is less sensitive than flux sampling to artifacts introduced by thermodynamically infeasible cycles and that its predictions are less susceptible to overfitting than EFA-based methods.

## Introduction

Genome-scale metabolic networks provide the basis for reconstructing the set of metabolic reactions occurring within a living organism. These reactions carry the flux of materials distributing the building blocks for macro-molecules production and, ultimately, biomass formation. However, flux configurations cannot be determined with complete certainty as experiments can only probe a fraction of all the states allowed by the various concentration levels that enzymes, RNAs, and metabolites can reach within a cell [1].

Alternatively, the mass balance principle can be applied to obtain a mathematical model describing the variation of all concentrations for the metabolic system. This model can be further simplified by assuming a steady-state condition, resulting in a linear mathematical model which provides a solution space for all possible flux configuration that comply with the constraints set by the stoichiometry of the reaction network. In this framework, metabolic networks can be considered as providing a space of possible flux configurations where some adaptive regulatory mechanism of the reaction rates resolves into the one that maximizes cellular fitness [2, 3]. An implementation of this idea has been Flux Balance Analysis (FBA) [4], which encodes known uptake rates and metabolites mass balances as constraints of a linear optimization problem where biomass growth rate is maximized. FBA has been an influential approach as sequencing technologies have allowed inferring the topology of metabolic networks at a genome-scale [5–7]. As FBA is formulated as a linear programming problem, it does not necessarily yield a single solution [8, 9]. This is typically the case for metabolic networks as they contain loops and alternative pathways [10] that accept various flux configurations to be compatible with a given set of know uptake rates and maximized objective function values [11]. Linear programming can be used to efficiently select a single flux configuration within this space, but its pick is based on the implementation of the algorithm performing the optimization rather than on biological considerations. This is problematic as different implementations may result in different solutions, which affect the reproducibility of results, and it can produce conflicting outcomes. For instance, two algorithmic implementations can predict flux through mutually exclusive pathways. Using alternative metrics of cellular fitness, e.g. ATP production, or measuring extra uptake rates is typically insufficient to reduce the solution space to a single flux configuration.

**Fig 1:**
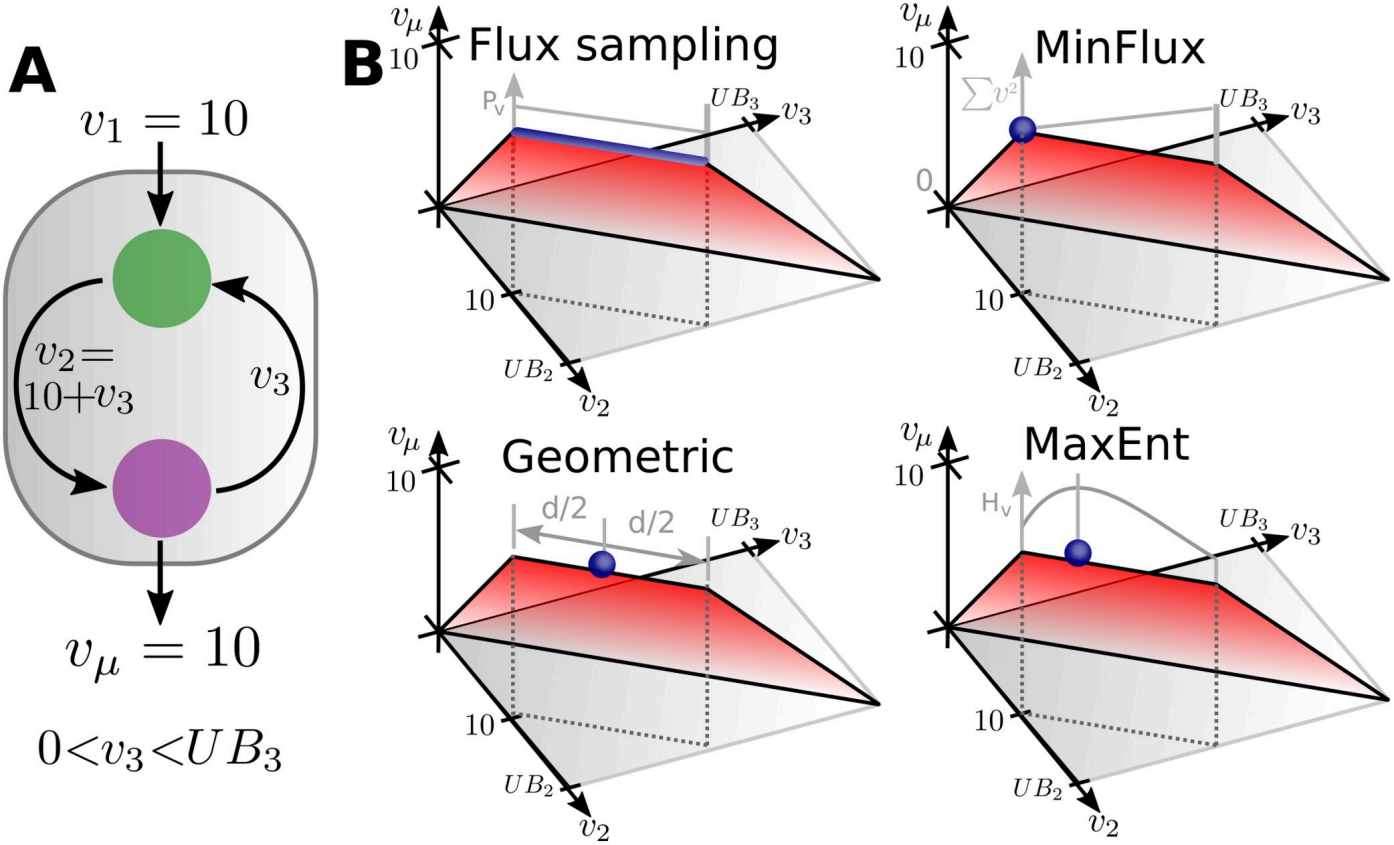
Polytope of possible flux configurations. (A) Example metabolic network with known exchange fluxes *v*_1_ and *v*_*μ*_, and two unknown inner fluxes *v*_2_ and *v*_3_ forming a loop. The upper bounds for fluxes *v*_2_ and *v*_3_ are *UB*_2_ and *UB*_3_. Any value of *v*_3_ ∈ [0, *UB*_3_] satisfy the metabolites’ mass balances, resulting in an infinite set of solutions. (B) Different methods to estimate the inner fluxes. Flux sampling estimates flux configurations from random samples from the set of alternative flux configurations (represented by the points forming the blue line). MinFlux selects the flux configuration where $v_{2}^{2}+v_{3}^{2}$ reach their minimum value according to the assumption that cells uses the the minimum amount of fluxes to economize enzyme synthesis. Geometric selects the flux configuration located at the geometric center of the space formed by the alternative solutions. MaxEnt selects flux configuration with the maximum information entropy (*H*_*v*_).

Various methods have been proposed to deal with the ambiguity of these alternative solutions. One of them is flux sampling, where a sequence of random samples from the space of alternative solutions is generated until the entire space is analyzed [12, 13]. Unlike FBA, flux sampling does not require defining an objective function [12]. This method has been applied to small catabolic networks [14] and to genome-scale networks [12] to infer the range and probability distribution values for each flux. However, the mass balances of the metabolic network are not enough to prevent thermodynamically infeasible flux cycles. For instance, in the example network presented in Fig 1, an arbitrarily large flux value can be cycled between the metabolites of the inner loop. Only the upper bounds imposed over these fluxes prevent them from reaching ever larger values. These bounds are not meant to be biologically meaningful so that the sampling space is arbitrarily biased. Considering each reaction Gibbs free energy can prevent thermodynamically infeasible cycles [15], but this information is not always available for each reaction of a genome-scale metabolic network. To circumvent this problem extra constraints can be added in order to rule out the formation of closed cycles [16] but this renders the problem computationally intractable at genome-scale [17]. As a consequence, the presence of thermodynamically infeasible loops remains an open problem that can severely bias the inferences drawn from flux sampling.

Alternatively, infeasible cycles can be avoided by considering only the subspace of alternative solution where the sum of all fluxes magnitudes reach their minimum [17]. In this subspace, reactions forming close loops attain flux values equal to zero, effectively preventing thermodynamically infeasible cycles. If fluxes magnitudes are measured by their squared values, the space of alternative solutions is reduced to a single flux configuration [18]. We will refer to this method as MinFlux. Alternatively, if fluxes magnitudes are measured by their absolute values, more than one flux configuration can achieve the same minimum sum of fluxes, rendering a subspace of alternative solutions. In this case, further assumptions can be made to select a single flux configuration from this subspace. In particular, Geometric is a constraint-based model that selects the flux configuration located at the geometric center of the polytope formed by these alternative solutions [19]. Regardless of how flux magnitudes are measured, the minimization of fluxes assumption is coherent with the hypothesis that under optimal growing conditions cells save energy by producing the minimum amount of enzyme-related proteins [20]. Compared to MinFlux, Geometric predicts flux configurations where more reactions have zero flux. This is a result of Geometric measuring fluxes magnitudes by their absolute values, which is a known sparsity inducing norm [21].

MinFlux and Geometric each yield reproducible results while avoiding thermodynamically infeasible cycles, but they also have limitations. On the one hand, it is not known if the minimization of fluxes’ assumption is universally valid. For instance, it has been observed in *Saccharomyces cerevisiae* and *Escherichia coli* that high glucose consumption rates are accompanied by the activation of otherwise shut off pathways, resulting in the production of overflow metabolites through non oxidative pathways [22–24]. Numerous explanations have been offered, including ATP savings for the production of non-oxidative enzymes (which by being smaller, compared to their oxidative counterparts, requires less ATP in their synthesis) [25, 26], limited uptake rates capacity [27], and an upper limit on the dissipation of Gibbs energy [28]. On the other hand, minimizing the sum of fluxes can break thermodynamically feasible cycles. For example, the isocitrate dehydrogenase reaction of the glyoxylate cycle would be systematically predicted to be inactive, although it is known to be active in *E*. *coli* [29–31]. Also, when a metabolite can be converted into another by more than one pathway, the minimization of the sum of the fluxes absolute values leads to the inactivation of all but one of these alternative pathways [17]. Thus, introducing the risk of overestimating the flux through the remaining active pathway.

Thus, current methods to estimate flux configurations are either overly sensitive to artifacts introduced by thermodynamically infeasible cycles or rely on assumptions that may not be universally valid. To overcome this, we propose to use statistical inference methods, specifically the principle of maximum entropy [32], which in general terms states that the best state of knowledge of a system –expressed as a probability distribution– is the one that admits the most ignorance besides prior information. This principle has been applied in biological sciences [33], including the metabolism of bacterial populations to infer statistical models from limited data. De Martino *et al*. (2016) [34] modeled the fluctuations of growth rates in *E*. *coli* using a Boltzmann probability distribution as this is the one that maximizes entropy under the constraint that the population’s average growth rate equals its experimental value. A sampling of the solution space of *E*. *coli* metabolic network based on Boltzmann distribution is proposed, producing distributions of growth rates that closely resemble experimental data. De Martino *et al*. (2018) [1] have applied this procedure to sample the space of flux configurations of the catabolic core of *E*. *coli* metabolism. This method produces flux distributions whose averages are closer to experimental data than those produced using FBA or uniform sampling. Fernandez de Cossio Diaz and Mulet (2019) [35] applied this approach to address cell-to-cell metabolic variability in Chinese hamster ovary cells population as a function of the dilution rate in a chemostat. Since the sampling procedure is intractable at genome-scale sized metabolic networks, the authors reduced the network by pruning reactions that do not carry flux when computed using FBA at various dilution rates. As FBA typically produces multiple alternative solutions, the authors selected the one where enzymatic costs are minimized. Tourigny (2020) [36] has expanded the application of these ideas, proposing that the maximum entropy principle can assign the best allocation of resources among elementary flux modes for maximizing expected return on investment of metabolic resources in the face of uncertain environmental conditions. As the number of elementary flux modes explodes with the network’s size [37], this approach was applied to a simplified model of yeast metabolism, reproducing the observed behavior of a cellular population in continuous and batch cultures.

Here, we use the principle of maximum entropy to determine a single flux configuration for a genome-scale metabolic network based on information theory. In particular, we proposed that the best estimation of the cellular flux configuration is the one with the minimum amount of unwarranted assumptions. Each flux configurations within the polytope of alternative solutions can be encoded as a probability distribution. The information entropy of these probability distributions can be interpreted as the average level of information inherent to each flux configuration. It follows that out of all flux configurations that are consistent with experimentally measured fluxes (for instance, glucose uptake), we should select the one with the largest value of information entropy [38], as it requires the fewest prior assumptions, and hence corresponds to the least biased solution. This idea was implemented at a genome-scale as a constraint-based model, which we called MaxEnt. MaxEnt finds the flux configuration with the most homogeneous distribution of fluxes that is consistent with the restrictions imposed by the constraint-based model. This makes MaxEnt less sensitive than flux sampling to the artifacts introduced by thermodynamically infeasible cycles as their fluxes are prevented from reaching their upper bounds. At the same time, MaxEnt predictions neither eliminate thermodynamically feasible cycles nor alternative pathways. The latter of which are biases introduced by MinFlux and Geometric methods.

In the methods section, we provide a formalism to apply information entropy to flux configurations. In the results section, we used this formalism to set up and test quantitative predictions for *E*. *coli* and *S*. *cerevisiae*. Here, we provide evidence that MaxEnt can improve our estimation of single flux configurations.

## Materials and methods

### General background

For a metabolic network of *N* reactions and *M* metabolites, we define the scalar vector of metabolites concentrations *c*, its time derivatives $\dot{c}$ and the scalar vector of fluxes *v*, where *c* and $\dot{c}\in R^{M}$, and $v\in R_{+}^{N}$. We have considered all fluxes to be non-negative, with reversible reactions been split-up into forward and reverse reactions. The stoichiometric matrix *S* has (*M* × *N*) elements, so that the product *Sv* results in a (*M* × 1) matrix. Thus, the time dependent mass balance is written as:

$$\begin{array}{c} \dot{c}=Sv \end{array}$$ (1)

Assuming steady-state condition for the metabolism, $\dot{c}=0$, and including further constraints for reversibility of reactions, uptake fluxes of nutrient, and kinetic limits in the form of lower $LB\in R_{+}^{N}$ and upper bounds $UB\in R_{+}^{N}$ on fluxes, a convex polytope P of alternative flux configurations is defined:

$$\begin{array}{c} P=\{v\in R_{+}^{N}|Sv=0,LB\leq v\leq UB\} \end{array}$$ (2)

Biomass growth rate is integrated into the metabolic network using a biomass reaction in the form of a linear combination of metabolic fluxes *v*_*μ*_ = ∑_*i*_ *b*_*i*_ *v*_*i*_, where *b*_*i*_ correspond to the mass proportion of the metabolite *i* in biomass.

### Genome-scale metabolic networks

Genome-scale metabolic networks reconstructions for *E*. *coli* and *S*. *cerevisiae* were used iJR904 (N = 1075 reactions, and M = 761 metabolites) [39] and iMM904 (N = 1577, M = 1226) [40], respectively. Both were obtained from the BiGG Models database [41].

### Information entropy modeling

Let us consider an experiment where reactions are randomly sampled from a metabolic network. The outcome of each sample can be encoded by the random variable *X* ∈ {*x*_1_, …, *x*_*N*_}, where *x*_*i*_ is the identity of reaction *i* (for instance the name of the enzyme catalyzing the reaction), and the probability of observing *x*_*i*_ is given by *P*_*v*_(*X* = *x*_*i*_). If *Q* enzymes are distributed among *N* reactions, such that *Q* = ∑_*j*_ *q*_*j*_, where *q*_*i*_ are the enzyme units catalyzing reaction *i*, then:

$$\begin{array}{c} P_{v}\left(X=x_{i}\right)=\frac{q_{i}}{\sum_{j}q_{j}} \end{array}$$ (3)

For reaction *i*, its flux *v*_*i*_ is a function of the amount of enzymes, namely *v*_*i*_ = *k*_*i*_ *η*_*i*_ *q*_*i*_, where *k*_*i*_ is the maximum turnover of catalyst *q*_*i*_ and *η*_*i*_ is a function ranging from 0 to 1 describing the decrease in catalytic rate due to intracellular conditions (for example, incomplete substrate saturation) [42]. Then, *q*_*i*_ in Eq 3 can be replaced by *v*_*i*_/(*k*_*i*_ *η*_*i*_). Unfortunately, values of *k*_*i*_ *η*_*i*_ are unknown for the vast majority of reactions [42], so that we assume their values to be similar to one another in order rewrite Eq 3 as:

$$\begin{array}{c} P_{v}\left(X=x_{i}\right)=\frac{v_{i}}{\sum_{j}v_{j}} \end{array}$$ (4)

This is an approximation that can be improved if values of *k*_*i*_ *η*_*i*_ became available. Still, each v∈P defines a different probability distribution for *X*, and the average level of information inherent to the various possible outcomes of *X* is given by its information entropy [38]:

$$\begin{array}{c} H_{v}\left(X\right)=-\sum_{i=1}^{N}P_{v}\left(X=x_{i}\right)\log P_{v}\left(X=x_{i}\right) \end{array}$$ (5)

*H*_*v*_(*X*) has two extreme values. Its minimum is 0 and is obtained when all but one flux in *v* are 0. In this case, we would be certain of the outcome of any random sample as *P*_*v*_(*x*_*k*_) = 1 for the non-zero flux, and 0 otherwise. On the other hand, the maximum value of *H*_*v*_(*X*) is obtained when all fluxes of *v* have the same value, generating a uniform probability distribution *P*_*v*_(*X*) = 1/*N*. In this case, *H*_*v*_(*X*) = log(*N*), which is the maximum uncertainty for the outcome of a random sample. These two limits are not biologically realistic but illustrate the notion that *H*_*v*_(*X*) corresponds to the average uncertainty contained in the outcome of this random sampling.

According to the principle of maximum entropy [32, 43], the v∈P that best represents our knowledge of the flux configuration of the cell is the one with the largest value of *H*_*v*_(*X*). *H* can be interpreted as the average number of yes/no questions that we would need to ask in order to determine the outcomes of *X* (when using two as the base of log). It follows that out of all v∈P, the one with the largest value of *H* should be selected, as this is the one that would require the fewest prior assumptions. Alternatively, the *v* that maximizes *H* can also be interpreted as the flux configuration that can happen in the greatest number of ways when a cell assigns a given amount of catalysts among its *N* reactions. This is the case if we assume that any two units of *Q* can be exchanged between reactions, for instance, by recycling the amino acids from one enzyme to produce another. Then, the greatest number of permutations in which these units of *Q* can be distributed among *N* reactions is given by the probability distribution that maximizes Boltzmann entropy [44], *S* = −*k*_*b*_∑_*i*_ *P*(*X* = *x*_*i*_)*log*(*P*(*X* = *x*_*i*_)), where *k*_*b*_ is the Boltzmann constant and *P*(*X* = *x*_*i*_) is the same as defined in Eq 4. Since argmax(*H*) = argmax(*S*) [45], it can be concluded that the v∈P maximizing *H* also allows the units of *Q* to be assigned in the greatest number of ways. Therefore, such *v* would be the most likely to be observed.

*H*_*v*_(*X*) is a strictly convex function [46], therefore there is only one v∈P that maximizes *H*_*v*_(*X*). Hence, we formulate MaxEnt as the following constraint-based problem:

$$\begin{array}{cc} & \max_{v}H_{v}\left(X\right)\\ \text{subject}\;\text{to}: & \\ & Sv=0\\ & LB\leq v\leq UB \end{array}$$ (6)

### Computational implementation

For the implementation, it is important to note that splitting reversible fluxes into non-negative forward and reverse fluxes introduces cycles, such as the one depicted in Fig 1A. These cycles admit an arbitrarily large amount of flux to be added to the forward and reverse fluxes and still produce a flux configuration compatible with the metabolites’ mass balances. This problem can be avoided by setting at least one flux to its experimentally observed value. Since MaxEnt finds the most uniform flux configuration that is compatible with the restrictions defined in Eq 6, the forward and reverse values of the unknown fluxes result in magnitudes similar to the experimentally known. For MaxEnt and all other methods, all reaction were assigned *LB* = 0 and *UB* = 1000, except for the biomass reaction, *v*_*μ*_, and exchange flux of glucose, *v*_*EX*_*glu*_, which were set to match their corresponding measured values: $LB_{EX\_glu}=UB_{EX\_glu}=v_{EX\_glu}^{Obs}$, and $LBv_{\mu }=UBv_{\mu }=v_{\mu }^{Obs}$.

All methods (MaxEnt, flux sampling, MinFlux, and Geometric) were implemented using the COBRApy 0.16.0 [47] library in Python 3.7. MaxEnt non-linear maximization was done using IPOPT 3.12.3 [48] optimizer through the CasADi 3.4.5 [49] interface. Flux sampling and Geometric were performed using the optGpSampler [13] and geometric_fba functions implemented in COBRApy. The minimization of MinFlux’s quadratic objective function was done using CPLEX 12.9.

To perform MaxEnt optimization via IPOPT we added a small number, *ϵ*, to each flux value in Eq 5 to avoid the undefined value of *LogP*_*v*_(*x*_*i*_) when *v*_*i*_ = 0. For all computations we used *ϵ* = 10^−8^. We used Flux Variability Analysis (flux_variability_analysis function of COBRApy) to narrow down the lower and upper bounds of each flux within the polytope of alternative solutions. Fluxes narrowed down to a single value were considered constants in MaxEnt, thus reducing the number of variables.

## Results

### Analysis of metabolic networks loops

**Fig 2:**
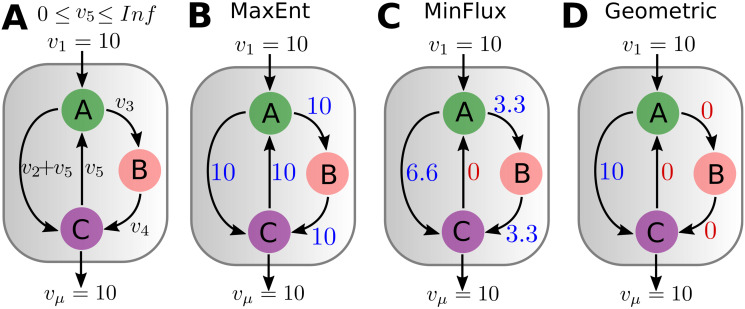
Example network with alternatives routes and a loop. (A) The metabolic network has a direct route from metabolites *A* to *C* and an indirect one mediated by metabolite *B*. A loop is formed by the reaction that goes from *C* back to *A*. The flux *v*_5_ can reach an arbitrarily large value without violating the metabolites’ mass balances. (B) Constrained by the uptake and production fluxes, MaxEnt predicts a network with all fluxes being equal to 10. Any deviation from this flux configuration results in a reduction in the value of *H*(*v*), thus ruling out arbitrarily large values of *v*_5_. (C) MinFlux predicts a zero flux value going from *C* to *A*. (D) Geometric also predicts zero flux from *C* to *A*, but as it measures fluxes’ magnitudes by their absolute values, it also predicts zero flux from the *A* to *B* and *B* to *C* reactions.

Loops are an important feature of metabolic networks. They have been proposed as essentials to explain the self-amplification capacity of metabolism and necessary to re-concentrate pathways’ inputs into a finite number of metabolic intermediates [50]. However, as information of the Gibbs free energy is not always available to determine the direction of reversible reactions, thermodynamically infeasible cycles can arise within the polytope of alternative solutions. This is illustrated by the example metabolic network presented in Fig 2A, which despite being constrained by its uptake (*v*_1_ = 10) and production (*v*_*μ*_ = 10) fluxes, can have an arbitrarily large flux value *v*_5_ cycling between metabolites *A* and *C*. Therefore, we started by analyzing how MaxEnt accounts for metabolic loops.

For the example network Fig 2A, MaxEnt yields a uniform distribution of fluxes (Fig 2B). By maximizing the information entropy, MaxEnt selects the most homogeneous configuration compatible with the observed fluxes, naturally tending to veer away from flux configurations where one or more fluxes have large value differences compared to the measured uptake and production fluxes. In this scenario, flux sampling would result in a distribution of values for *v*_5_ that would only be bounded by the upper limit imposed on this flux, which in itself is not biologically meaningful.

**Fig 3:**
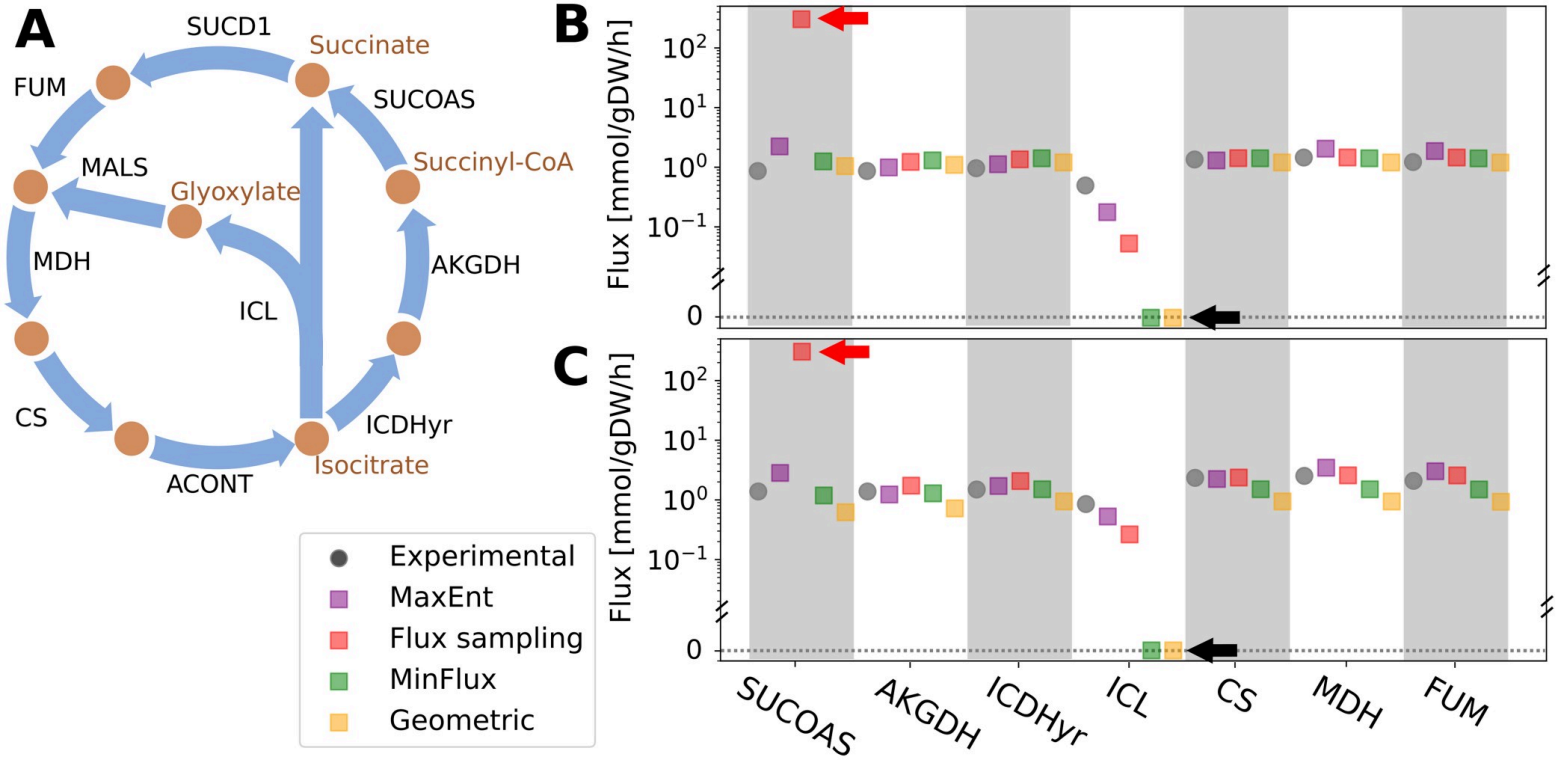
*E*. *coli* Krebs cycle and glyoxylate shunt. (A) The glyoxylate shunt is a two-step metabolic pathway (isocitrate lyase, ICL; and malate synthase, MALS) that bypasses the Krebs cycle carbon dioxide-producing steps [56]. As it forms a loop, the economy of fluxes assumption would predict no flux through it, which contradicts experimental data. (B) and (C) show the predicted and experimental fluxes of the glyoxylate shunt and Krebs cycle at two specific biomass growth rates, 0.1 [1/h] and 0.2 [1/h], respectively. In both cases, MaxEnt and flux sampling correctly predict flux through ICL. However, flux sampling predicts a flux magnitude through succinyl-CoA synthetase (SUCOAS) which is two orders of magnitude above the observed value (red arrows). On the other hand, MinFlux and Geometric predict zero flux through ICL, see black arrows. For flux sampling, the values reported correspond to the median of 1,350,000 samples and thinning = 1000 (see S1 and S2 Figs).

On the other hand, MinFlux and Geometric can avoid the artificially large flux values of *v*_5_ as they select flux configurations with the minimum sum of fluxes (Fig 2C and 2D). Although it is plausible to assume that cells minimize the energy costs associated with the production of the enzymes carrying out the metabolic reactions, it is not clear if these fluxes should be zero. A known case is isocitrate lyase (ICL) reaction, which in *E*. *coli* creates a nested cycle within the Krebs cycle, the glyoxylate shunt (see Fig 3A). This reaction has been observed to have positive flux [29–31].

To test if MaxEnt would produce non zero flux value through *E*. *coli*’s glyoxylate shunt at a genome-scale level, we compared its predictions to 25 measured fluxes including ICL [29–31, 51–54] at two growth rates (data was retrieved from CeCaFBD [55], see also S1 File). To have a reference point, we also computed the flux configuration using flux sampling (1,350,000 samples taken within the space P), and the single predictions of MinFlux and Geometric. The results, presented in Fig 3B and 3C, show that only MaxEnt and flux sampling predict flux through the glyoxylate shunt. Comparing to experimental fluxes of the Krebs cycle, MaxEnt predicted values in the same order of magnitude (Fig 3B and 3C). On the contrary, flux sampling predicts an average SUCOAS flux that is two orders of magnitude above the experimental result, being this the result of flux sampling not able to rule out thermodynamically infeasible flux values [17]. On the other hand, MinFlux and Geometric were unable to predict flux through the glyoxylate shunt as expected by their underlying economy of fluxes assumption.

**Fig 4:**
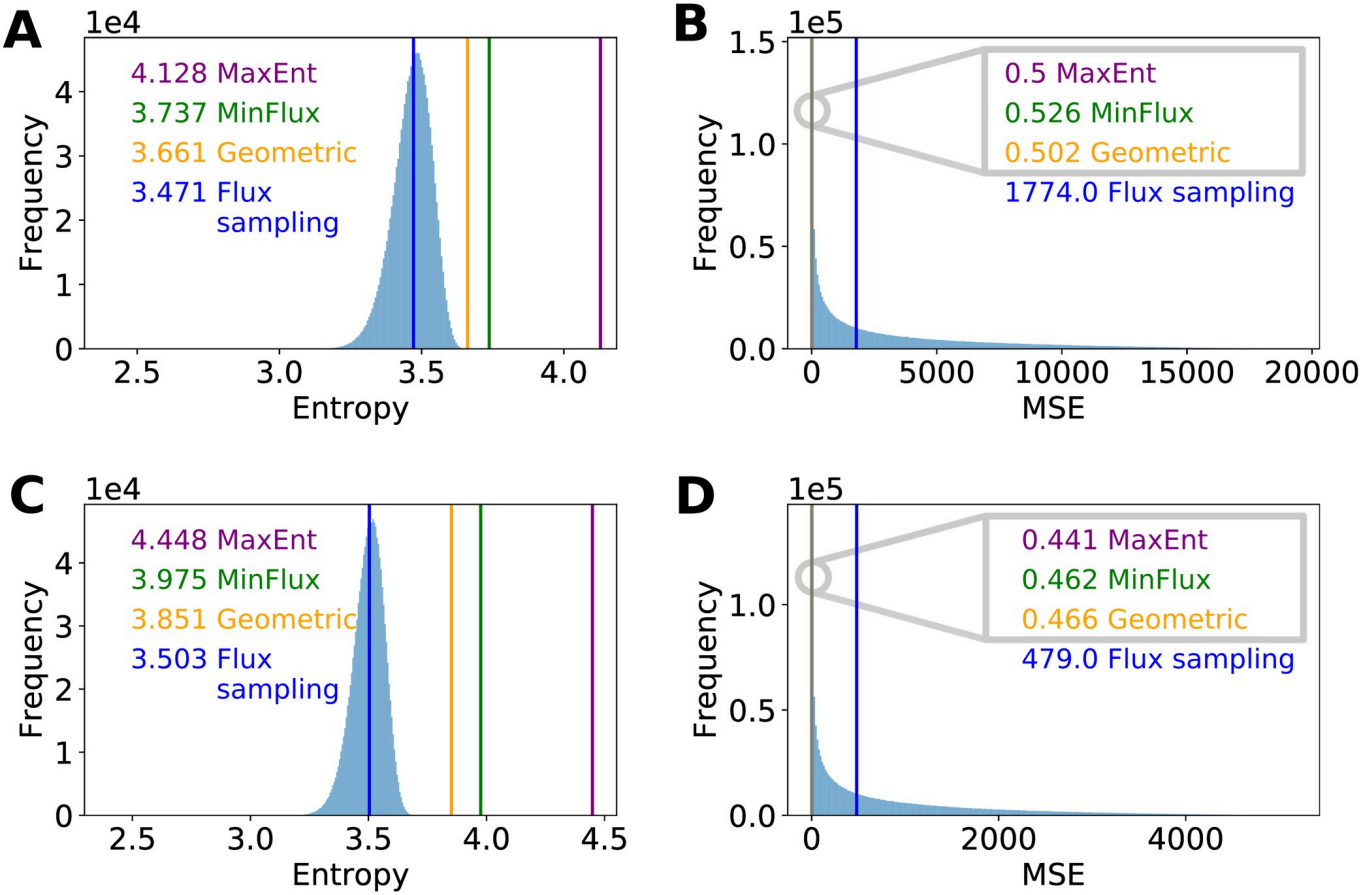
Entropy and MSE at two *E*. *coli* growth rates. (A) and (B) show the entropy and MSE values at a growth rate of 0.1 [1/h]. (C) and (D) show the same indices at a growth rate of 0.2 [1/h]. For both growth rates, MaxEnt predictions have a statistically significantly larger information entropy (*p*-values < 10^−5^, one-tailed test of a normal distribution) compared to the median entropy of flux sampling, with the entropy of MinFlux and Geometric predictions falling between them. MaxEnt predictions have an MSE value in the same order of magnitude as the ones obtained by MinFlux and Geometric, but at least three orders of magnitude lower than the median MSE of flux sampling, supporting MaxEnt capacity to predict inner metabolic fluxes. For flux sampling, 1,350,000 samples from the space of alternative solutions (thinning = 1000) were taken, and for each sample, entropy and MSE were computed. The resulting distributions are shown in light blue.

To analyze if current methods already produced flux configurations with high information entropy levels, we determined their information entropy by using Eqs 4 and 5 to their predictions (in the case of flux sampling, we used the average flux values). We found that MaxEnt predictions (Fig 4A and 4C) have a significantly larger information entropy (*p*-values < 10^−5^, one-tailed test of a normal distribution) than the mean information entropy obtained by flux sampling, with MinFlux and Geometric having information entropy values in between these two methods.

We further studied MaxEnt predictions by comparing them to the rest of the 25 experimentally observed metabolic fluxes, which span the central catabolic core of *E*. *coli*. We quantified the similarity between predicted and measured fluxes using mean-squared error (MSE):

$$\begin{array}{c} MSE=\frac{1}{N_{f}}\sum_{i=1}^{N_{f}}{\left(\frac{v_{i}}{\left|v_{Ex\_glu}\right|}-\frac{v_{i}^{Obs}}{\left|v_{Ex\_glu}^{Obs}\right|}\right)}^{2}, \end{array}$$ (7)

where the measured $v_{i}^{Obs}$ and predicted *v*_*i*_ flux are normalized by the measured $|v_{Ex\_glu}^{Obs}|$ and predicted $|v_{Ex\_glu}^{Obs}|$ magnitude of the exchange glucose rate.

We found that MaxEnt, MinFlux, and Geometric outperforms the average solution of flux sampling (Fig 4), producing MSE values that are more than 3 orders of magnitude lower than the median MSE of flux sampling (Fig 4B and 4D). This suggests that the accuracy of flux sampling predictions is highly sensitive to artifacts introduced by thermodynamically infeasible cycles.

**Fig 5:**
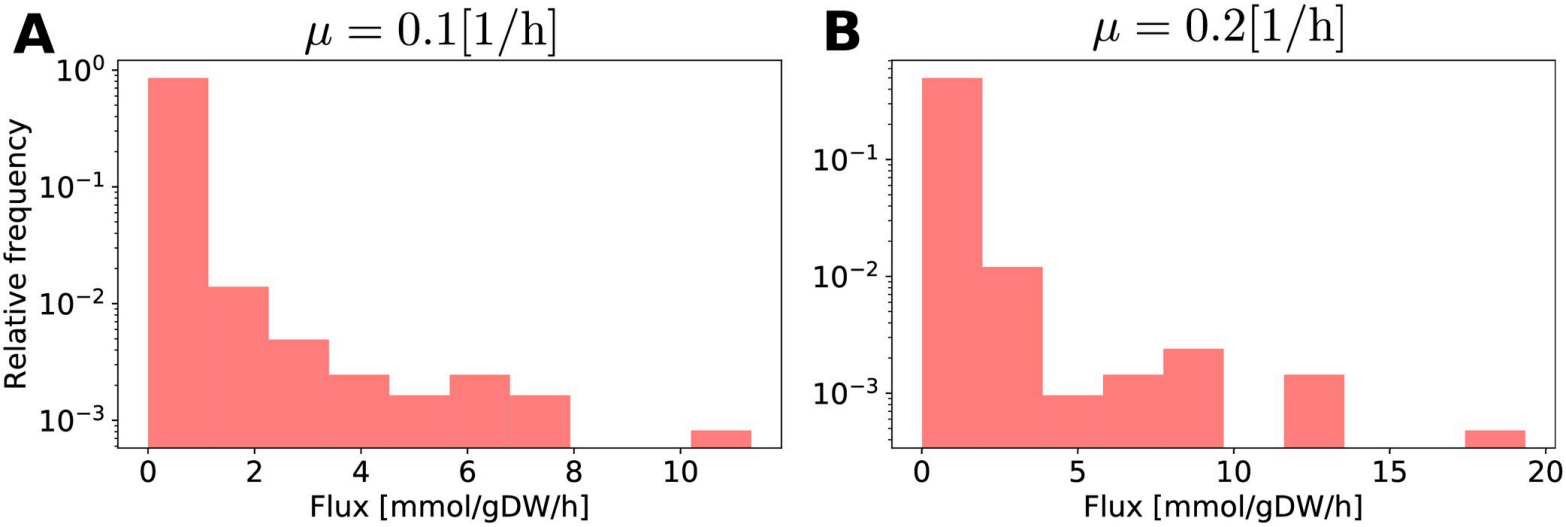
Relative frequency of fluxes estimated by MaxEnt for *E*. *coli*. (A) and (B) show histograms of fluxes of the solution of MaxEnt at growth rates 0.1 and 0.2 [1/h] in *E*. *coli*. For both histograms 10 bins were used.

Previous results have shown that *E*. *coli* fluxes are distributed according to a power-law distribution [57], with most reactions having zero flux and only a few of them having large flux values. On the other hand, MaxEnt finds the most uniform distribution of fluxes compatible with the optimization problem’s restrictions defined in Eq 6. To verify whether MaxEnt formulation produces a rich and structured distribution of fluxes or not, histograms of the flux values for *E*. *coli* predicted by MaxEnt at growth rates of 0.1 and 0.2 [1/h] are presented in Fig 5A and 5B. These results show that MaxEnt solution follows a power-law distribution, which was verified by plotting the same results in a log-log scale (see S3 Fig).

### Predicting flux configuration considering overflow metabolism

MaxEnt, MinFlux, and Geometric produced flux configuration predictions with low MSE at specific growth rates of 0.1 and 0.2 [1/h], suggesting that under these growing conditions, they can predict the fluxes of the central carbon system. However, it has been observed in *S*. *cerevisiae* and *E*. *coli* that high glucose uptake rates are accompanied with partial oxidation pathways, resulting in the production of overflow metabolites: acetate, ethanol and lactate, respectively [22–24]. As a result, overflow metabolism produces a redistribution of fluxes through previously inactive pathways, which is at odds with the economy of fluxes assumption underlying MinFlux and Geometric.

**Fig 6:**
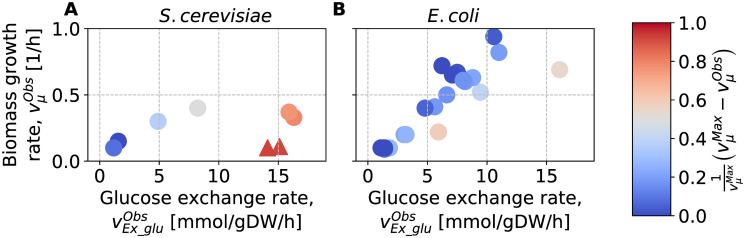
Metabolism of *S*. *cerevisiae* and *E*. *coli* data at various levels of overflow metabolism. We estimated the level of overflow metabolism as the normalized difference between observed $v_{\mu }^{Obs}$ and theoretical maximum $v_{\mu }^{Max}$ biomass growth rates. (A) Various growth conditions of *S*. *cerevisiae* are represented by their observed rates of glucose exchange (uptake) and biomass growth. Δ correspond to mutated strains overproducing acetate. (B) Various growth conditions of *E*. *coli*.

To investigate if MaxEnt is able to produce reasonable predictions at various levels of overflow metabolism, we compared its predictions against a set of 8 growth conditions in *S*. *cerevisiae* [58–61], and a set of 25 growth conditions in *E*. *coli* [29, 30, 52–54, 62–74] (data was retrieved from CeCaFBD [55], see also S2 File). In each set, the data-points vary in terms of both the uptake rate of glucose and biomass growth rate. FBA is a good predictor for the growth rate in the absence of overflow metabolism, but it overestimates the growth rates otherwise. We took advantage of this to quantify the level of overflow metabolism in the experimental data by measuring the difference between the maximum theoretical growth rate (as computed by FBA) and the actual growth rate, with the difference normalized by the maximum theoretical growth rate. The results (see Fig 6) show that the datasets of *S*. *cerevisiae* and *E*. *coli* span growth conditions with various levels of overflow metabolism.

**Fig 7:**
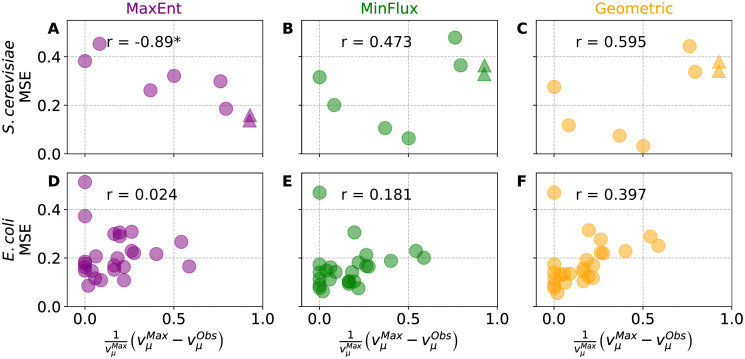
Correlation between overflow metabolism and Mean Square Error (MSE). (A), (B), and (C) show *S*. *cerevisaie*’s MSE between observed and predicted inner metabolic fluxes. MinFlux and Geometric predictions outperform MaxEnt when the level of overflow metabolism is below 0.5 but thereafter the situation inverts, suggesting that the minimization of fluxes assumption of MinFlux and Geometric may not be universally suitable. (D), (E), and (F) show a similar trend in *E*. *coli*. MinFlux and Geometric predictions have lower MSE than MaxEnt at low levels of overflow metabolism, but their MSE shows a positive Pearson correlation, *r*, at higher levels of overflow metabolism. On the other hand, MaxEnt shows a close to zero correlation with the level of overflow metabolism in both species. * indicates statistically significant Pearson correlation values (*p*-value ≤ 0.005). Data points are color coded as • MaxEnt, • Geometric, and • MinFlux. Δ correspond to mutated strains overproducing acetate.

Then, for *S*. *cerevisiae* and *E*. *coli*, we set their specific growth and glucose uptake rates to match their observed values and used MaxEnt to predict the corresponding flux configurations. The results (see Fig 7) show that MinFlux and Geometric predictions have lower MSE values compared to MaxEnt when the level of overflow metabolism is close to zero, but that the situation reverts at levels of overflow metabolism close to 1. On the contrary, MaxEnt prediction performance seems unaffected by higher levels of overflow metabolism. Although not statistically significant, these results support the use of MaxEnt over MinFlux and Geometric when metabolic pathways deviate from biomass production to generation of overflow metabolites.

There has been extensive work to explain the simultaneous use of ATP-efficient and inefficient pathways during overflow metabolism [75]. Several constraint-based models have been able to reproduce overflow metabolism behavior [76] by integrating relevant information coming from proteomics [77], gene expression [78], limitation in oxygen uptake rates [79], and free energy dissipation [28]. The extra information used by these models reduces the solution space but typically does not single out a unique flux configuration. As a constraint-based model, MaxEnt can be swiftly integrated into these models, helping the study of overflow metabolism by estimating a single flux configuration without adding extra assumptions.

**Fig 8:**
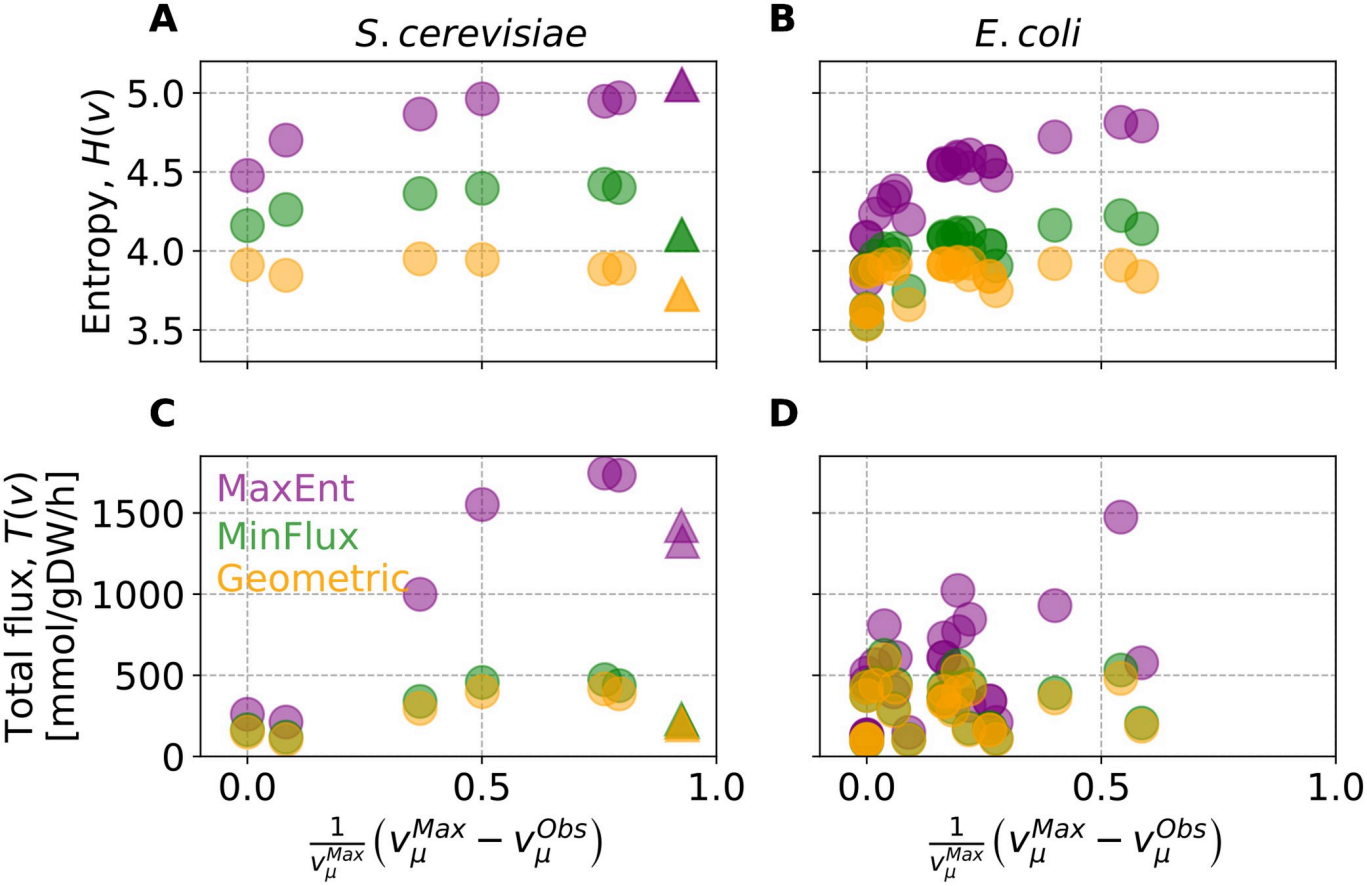
Information entropy and flux though the metabolic network predicted by various methods. (A) and (B) show the information entropy of the flux configurations for *S*. *cerevisiae* and *E*. *coli*, respectively. For each time point, MaxEnt predictions always have greater information entropy compared to MinFlux and Geometric. (C) and (D) show the total flux of the metabolic configurations predicted for *S*. *cerevisiae* and *E*. *coli*, respectively. MaxEnt predicts larger total flux compared to the other two methods, as the later rely on the minimization of fluxes assumption. Data points are color coded as • MaxEnt, • Geometric, and • MinFlux. Δ corresponds to mutated strains overproducing acetate.

To explore the differences between MaxEnt, on the one hand, and MinFlux and Geometric, on the other, that could explain their divergent behavior at higher levels of overflow metabolism, we analyzed the information entropy of their predictions. The results (see Fig 8A and 8C) show that the information entropy increases with the level of overflow metabolism. This likely stems from the additional metabolic fluxes activated to divert flux from biomass to produce overflow metabolites. To test this, at each growth condition, we measured the total flux:

$$\begin{array}{c} T\left(v\right)=\sum_{i=1}^{N}v_{i} \end{array}$$ (8)

We found that all methods increase their total flux with overflow metabolism (see Fig 8C and 8D), forming a saturation curve as the level of overflow metabolisms increases. Compared to MinFlux and Geometric, MaxEnt predicts larger increments in total flux, this being coherent with its tendency to predict a more homogeneous distribution of fluxes, and it results in more reactions carrying flux.

### Computing times

**Fig 9:**
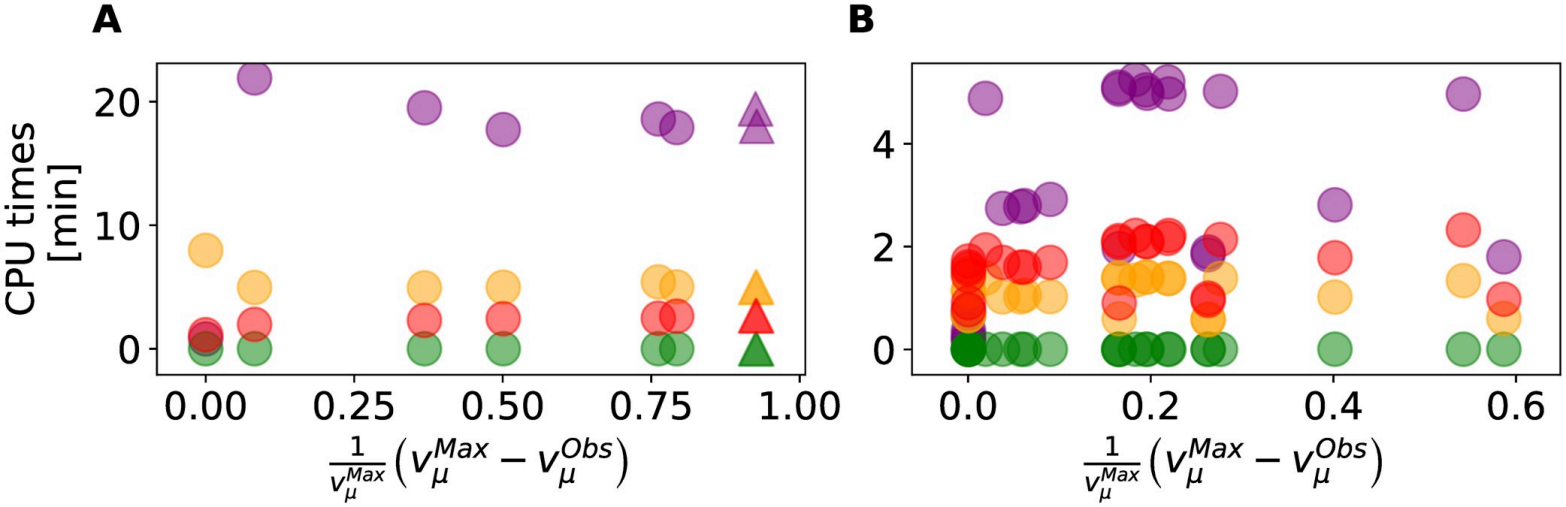
CPU times for various levels of overflow metabolism. (A) iMM904 (*Saccharomyces cerevisiae*), and (B) iJR904 (*Escherichia coli*). Data points are color coded as • MaxEnt, • Geometric, • flux sampling and • MinFlux. Δ corresponds to mutated strains overproducing acetate. 1000 samples were used in flux sampling.

Finally, we compared MaxEnt and alternative methods CPU times for various levels of overflow metabolism. The results (see Fig 9) show that the CPU times of MaxEnt, Geometric, and flux sampling are all within the same order of magnitude. Only MinFlux resulted in CPU times within fractions of a second. MaxEnt CPU times do not increase linearly with the level of overflow metabolism but caps on average at 20 min for the iMM904 network of *S*. *cerevisiae* and 6 min for the iJR904 network of *E*. *coli*. MaxEnt was implemented using out of the shelve algorithms, and its CPU times may be further reduced if a tailored implementation is used.

## Conclusion

Given a set of measured fluxes, constraint-based models typically predict a consistent space of flux configurations. In this work, we present a method based on the principle of maximum entropy, which in this context states that the best estimation of fluxes is the one with the least amount of unwarranted assumptions. We searched for the least bias flux configuration by computing its information entropy. Based on this, we formulated a constraint-based approach, MaxEnt, to find a single flux configuration that maximizes the information entropy within the space of alternative solutions. We found that MaxEnt predictions avoid artificially large flux values due to thermodynamically infeasible cycles in the metabolic networks. MaxEnt correctly predicted flux through the ICL reaction of the glyoxylate shunt of *E*. *coli*, which the alternative methods, MinFlux and Geometric, missed as they systematically avoid the formation of cycles. Unlike flux sampling, MaxEnt predicts fluxes in the same order of magnitude as the experimentally observed ones. MaxEnt also produces accurate estimations of the fluxes of the central carbon systems of *E*. *coli* and *S, cerevisiae* at various levels of overflow metabolism. In all these cases, MaxEnt does not require prior assumptions about the distribution of fluxes or their bounds, both of which can introduce observer bias in the results. By selecting the least bias flux configuration, MaxEnt is less prone to over-fitting, which is its main advantage over alternative methods, and may prove useful for estimating flux configurations when there is not sufficiently available *bona fide* information to constraint the solution space to a single point.

## Supporting information

S1 Fig Flux value distributions for reactions of the Krebs cycle and glyoxylate shunt at growth rate 0.1 [1/h]. For each reaction, a distribution of 1,350,000 flux values was obtained using flux sampling (thinning = 1000).
(TIF)

S2 Fig Flux value distributions for reactions of the Krebs cycle and glyoxylate shunt at growth rate 0.2 [1/h]. For each reaction, a distribution of 1,350,000 flux values was obtained using flux sampling (thinning = 1000).
(TIF)

S3 Fig Frequency of the flux configuration values predicted by MaxEnt for *E*. *coli* at growth rates 0.1 and 0.2 [1/h]. The figures are scatter plots in log-log scale.
(TIF)

S1 File Flux data for *E*. *coli* at growth rates 0.1 and 0.2 [1/h]. (XLS)

S2 File Flux data for *S*. *cerevisiae* and *E*. *coli* at various growth conditions. (XLSX)
