# Supplementary figures and images for: Metabolic flux configuration determination using information entropy
Source: PLoS One. 2020 Dec 4;15(12):e0243067. doi: 10.1371/journal.pone.0243067 (PMC7717585; doi:10.1371/journal.pone.0243067)

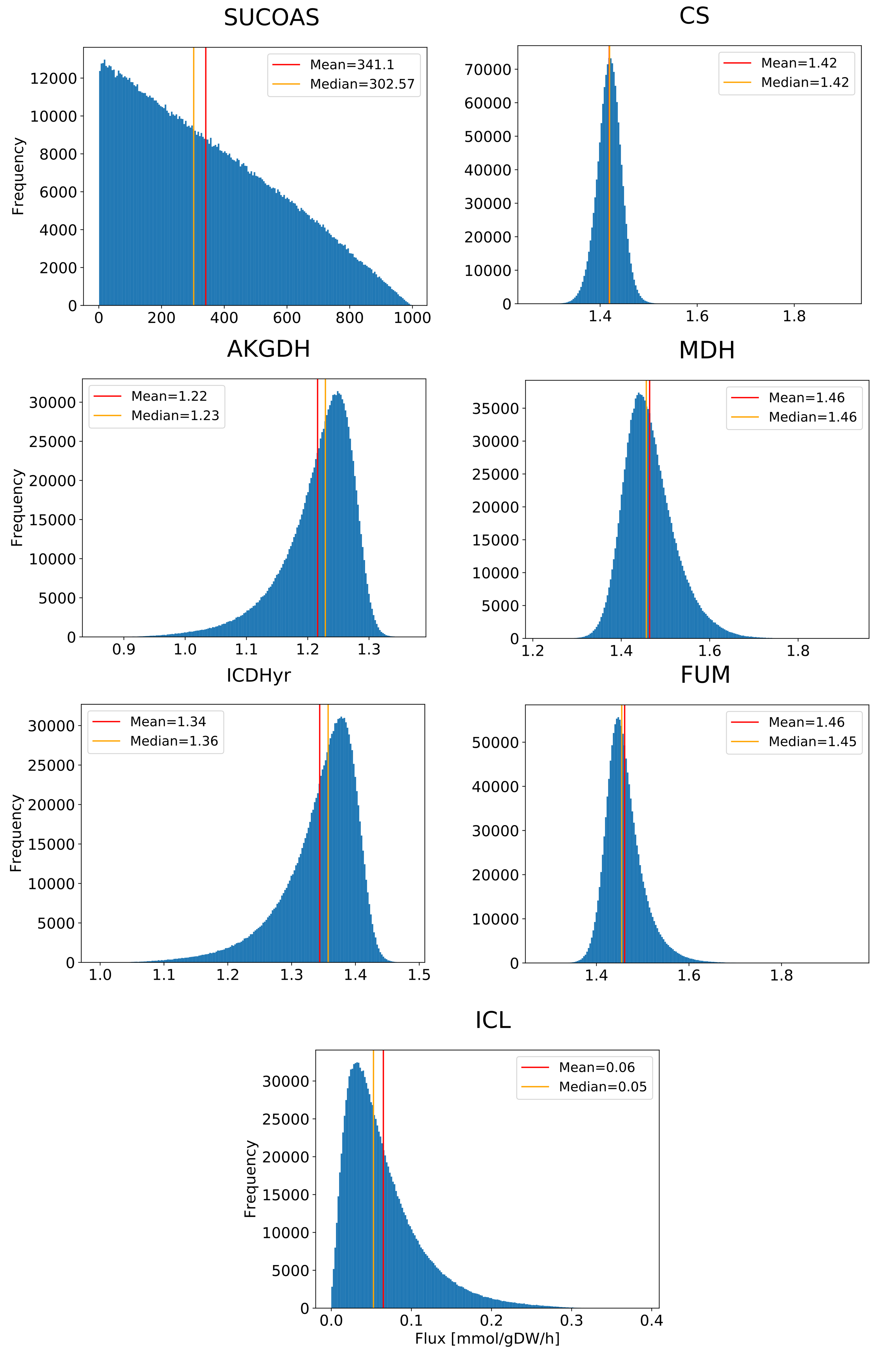

Supplement: S1 Fig — For each reaction, a distribution of 1,350,000 flux values was obtained using flux sampling (thinning = 1000). (TIF) [file pone.0243067.s001.tif]

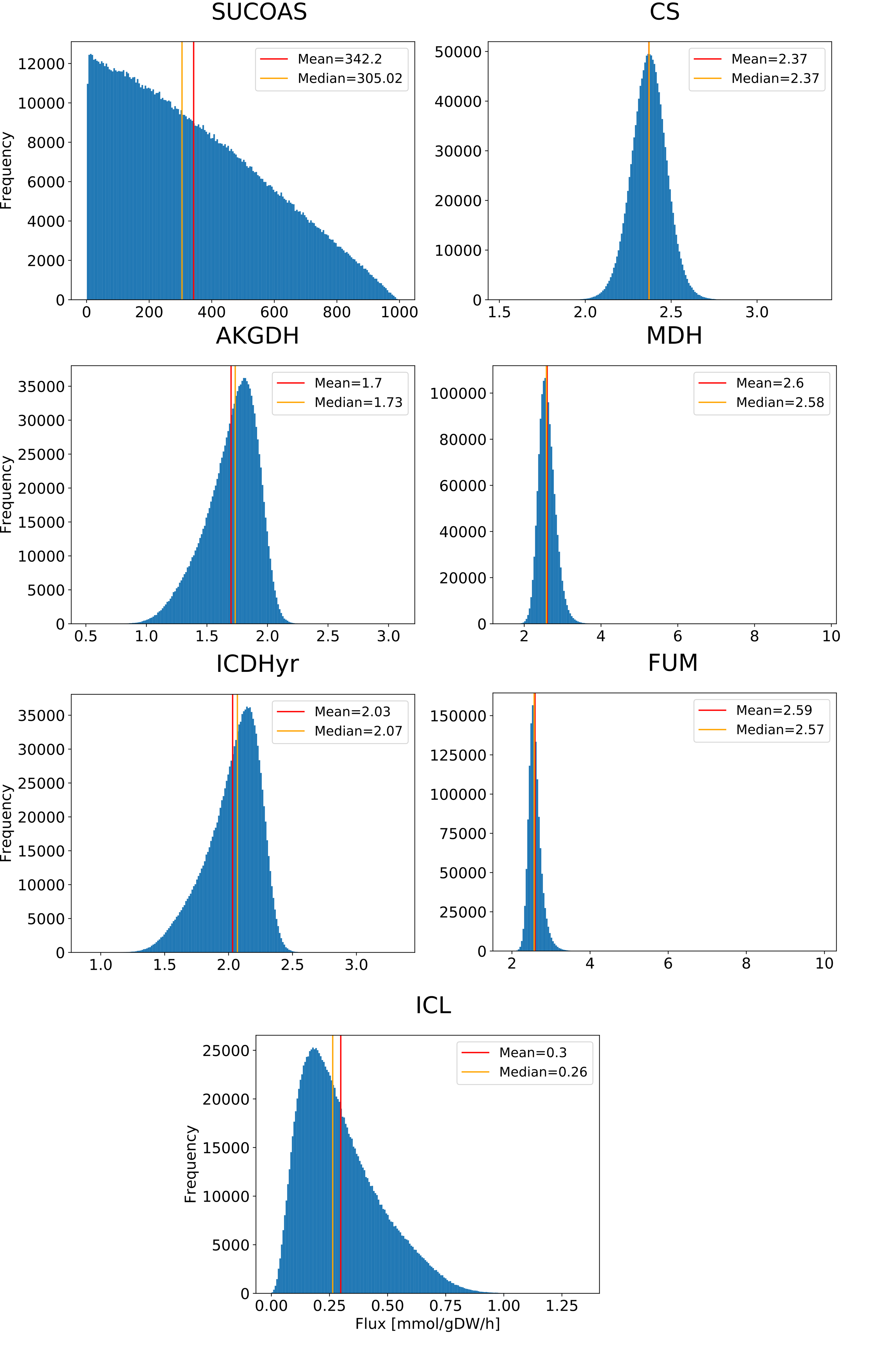

Supplement: S2 Fig — For each reaction, a distribution of 1,350,000 flux values was obtained using flux sampling (thinning = 1000). (TIF) [file pone.0243067.s002.tif]

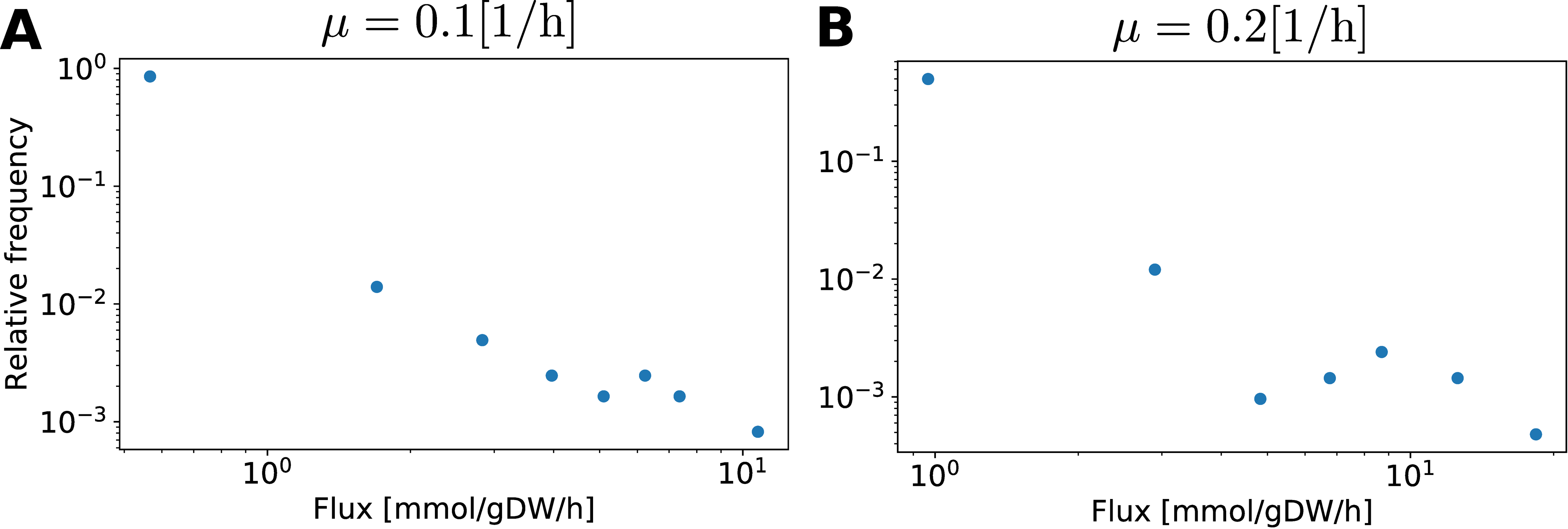

Supplement: S3 Fig — The figures are scatter plots in log-log scale. (TIF) [file pone.0243067.s003.tif]
